# Operative treatment of fragility fractures of the pelvis: a critical analysis of 140 patients

**DOI:** 10.1007/s00068-021-01799-6

**Published:** 2021-10-11

**Authors:** Pol Maria Rommens, Alexander Hofmann, Sven Kraemer, Miha Kisilak, Mehdi Boudissa, Daniel Wagner

**Affiliations:** 1 Department of Orthopedics and Traumatology, University Medical Center, Johannes Gutenberg-University, Langenbeckstrasse 1, 55131 Mainz, Germany; 2 Department of Orthopedics and Traumatology, Westpfalz Clinics Kaiserslautern, Helmut-Hartert-Strasse 1, 67655 Kaiserslautern, Germany

**Keywords:** Pelvis, Fragility fracture, Operative, Open, Percutaneous, Complications, Mortality, Outcome

## Abstract

**Background:**

Fragility fractures of the pelvis (FFP) are a clinical entity with an increasing frequency. Indications for and type of surgical treatment are still a matter of debate.

**Purpose:**

This retrospective study presents and critically analyses the results of operative treatment of 140 patients with FFP.

**Setting:**

Level-I trauma center.

**Materials and methods:**

Demographic data, comorbidities, FFP-classification, type of surgical stabilization (percutaneous (P-group) versus open procedure (O-group)), length of hospital stay (LoS), general in-hospital complications, surgery-related complications, living environment before admission, mobility and destination at discharge were retracted from the medical and radiographic records. Patients were asked participating in a survey by telephone call about their quality of life. SF-8 Physical Component Score (PCS) and SF-8 Mental Component Score (MCS) were calculated as well as the Parker Mobility Score (PMS) and the Numeric Rating Scale (NRS).

**Results:**

Mean age was 77.4 years and 89.3% of patients were female. 92.1% presented with one comorbidity, 49.3% with two or more comorbidities. Median length of hospital stay was 18 days, postoperative length of hospital stay was 12 days. 99 patients (70.7%) received a percutaneous operative procedure, 41 (29.3%) an open. Patients of the O-group had a significantly longer LoS than patients of the P-group (*p* = 0.009). There was no in-hospital mortality. There were significantly more surgery-related complications in the O-group (43.9%) than in the P-group (19.2%) (*p* = 0.006). Patients of the O-group needed more often surgical revisions (29.3%) than patients of the P-group (13.1%) (*p* = 0.02). Whereas 85.4% of all patients lived at home before admission, only 28.6% returned home at discharge (*p* < 0.001). The loss of mobility at discharge was not influenced by the FFP-classes (*p* = 0.47) or type of treatment (*p* = 0.13). One-year mortality was 9.7%. Mortality was not influenced by the FFP-classes (*p* = 0.428) or type of treatment (*p* = 0.831). Median follow-up was 40 months. SF-8 PCS and SF-8 MCS were moderate (32.43 resp. 54.42). PMS was 5 and NRS 4. Follow-up scores were not influenced by FFP-classes or type of treatment.

**Conclusion:**

Patients with FFP, who were treated operatively, suffered from a high rate of non-lethal general, in-hospital complications. Open surgical procedures induced more surgery-related complications and surgical revisions. Mental and physical follow-up scores are low to moderate. Condition at follow-up is not influenced by FFP-classes or type of treatment. Indications for operative treatment of FFP must be critically examined. Surgical fixation should obtain adequate stability, yet be as less invasive as possible. The advantages and limitations of different surgical techniques have to be critically evaluated in prospective studies.

## Introduction

**Fig. 1:**
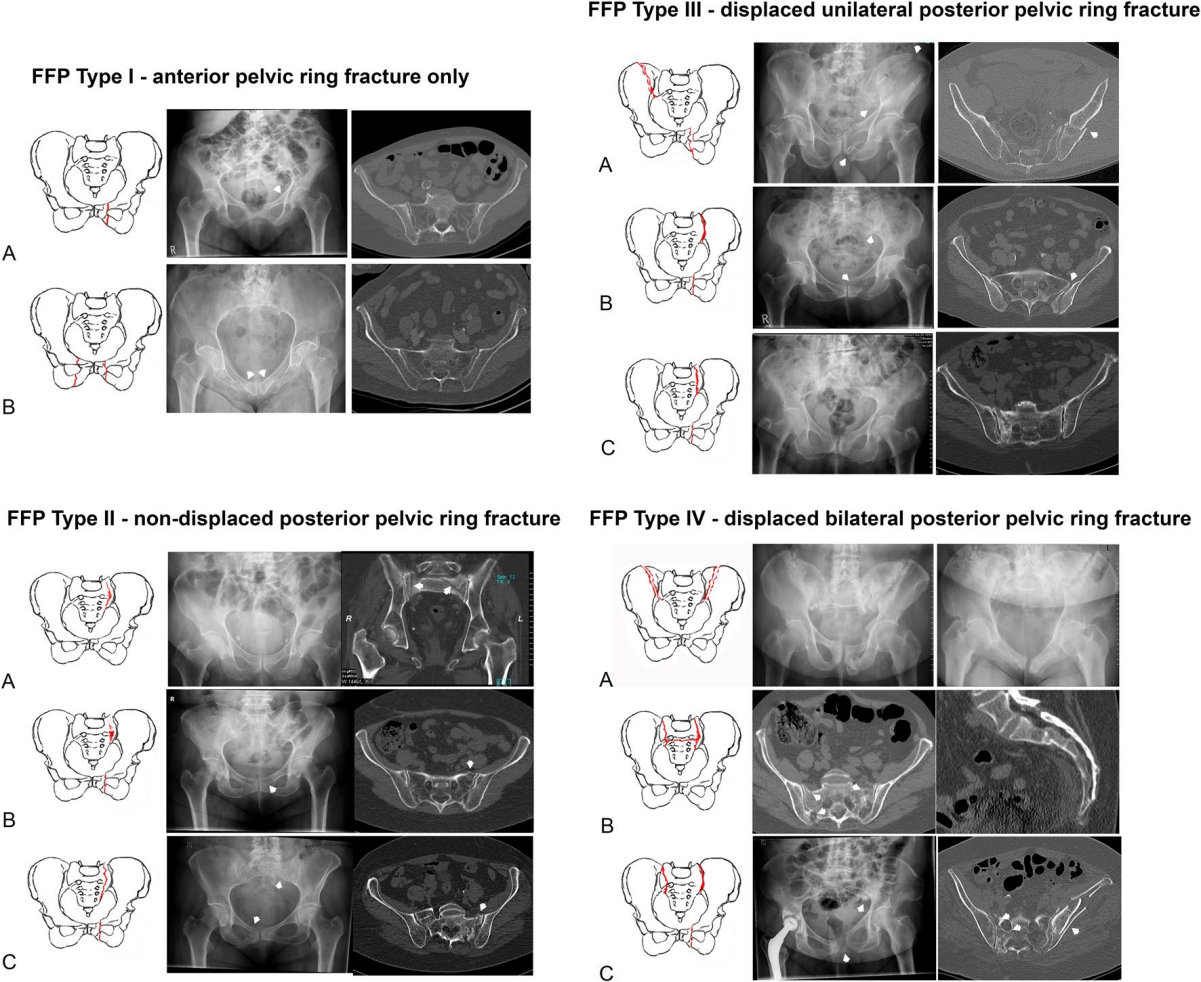
Classification of FFP in accordance with Rommens and Hofmann [6]

Fragility fractures of the pelvis (FFP) represent an increasing entity among low-energy fractures of elderly persons [1–3]. Patients suffering from FFP are very old; the vast majority of them are female. They declare intense, immobilizing pain in the pelvic region. A fall from sitting or standing position in domestic environment is the most frequent trauma mechanism. Sometimes, a traumatic event is not memorable. Many patients are admitted in the emergency department shortly after the fall, others present later with persistent complaints in the groin or gluteal region. FFP are detected by conventional pelvic radiographs. Due to low bone mineral density and superposition of bowel gases and intestinal contents, assessment of the posterior pelvis is mostly difficult. CT examination is imperative for complete analysis. Axial, coronal and sagittal reconstructions enable a detailed investigation of bony and surrounding soft tissue structures. With dual-energy-CT, the sensitivity for the presence of occult fractures is equal to MRI and higher than conventional CT [4, 5]. FFP exhibit other characteristics than high-energy pelvic fractures. A specific classification was developed, which distinguishes between different categories of instability. This classification is based on the assessment of conventional X-rays and CT. FFP type I include fractures of the anterior pelvis only. FFP type II involve non-displaced fractures of the posterior pelvis, FFP type III displaced unilateral and FFP type IV displaced bilateral fractures of the posterior pelvis (Fig. 1) [6, 7].

Treatment of FFP is focused on pain relief and as early mobilization as possible. Patients with FFP are of old age and suffer from accompanying illnesses, which put them at risk for complications and enhanced mortality [8, 9]. Any management should, therefore, be less invasive, include the amelioration of the general condition and the prevention of further fragility fractures [10]. Non-operative treatment brings dangers of bedridden conditions such as pneumonia and urinary tract infection. Operative treatment is associated with surgical complications like haematoma and surgical site infection. The fragile bone of elderly persons enhances the risk of implant loosening with loss of stability of the bone–implant construct. To date, there is no consensus on indications for and type of surgical treatment of FFP. Several authors describe large series of patients with reasonable results after non-operative treatment [9, 11, 12], whereas other authors report on good outcome after operative treatment [13–17]. This manuscript presents and critically analyses the results of operative treatment of 140 patients with FFP, depending on FFP-classification and invasivity of surgical treatment.

## Materials and methods

We retrospectively reviewed the medical charts and available radiographs of all adult patients, who were admitted with a pelvic fracture at the Department of Orthopedics and Traumatology of the University Medical Center Mainz, Germany, between 2005 and mid-2018 (13.5-year period). Excluded were patients with pelvic fractures after high-energy trauma and patients with acetabular fractures. Excluded were also patients with low-energy pelvic fractures, which were treated non-operatively. Included were only patients with an FFP, who underwent surgical treatment in our Department. A flowchart of excluded and included patients is presented in Table 1. The fracture patterns of the included patients were classified in accordance to the FFP-classification of Rommens and Hofmann [6]. Operative treatment was recommended to all patients with FFP type III and type IV and to patients with FFP type II after failed non-operative treatment (persistent immobilizing pain one week after admission, fracture progression). Painful non-union in patients with FFP type I was also seen as an indication for operation [7, 18]. All patients were operated after written informed consent. When patients had no mental capacity for approval, their legal representatives signed the documents.

**Table 1:**
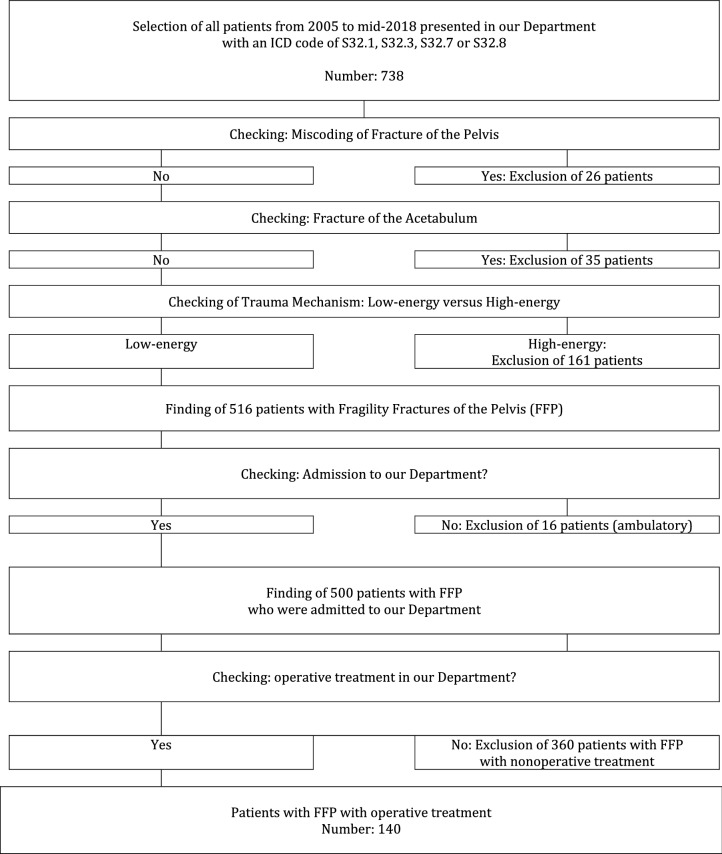
Flowchart of included and excluded patients

The following demographic data were collected: age, sex, comorbidities at admission (cardiovascular or pulmonary disease, diabetes mellitus, dementia, osteoporosis, rheumatoid arthritis, malignancy). Furthermore, the medical charts were analysed for the following information: total and postoperative length of hospital-stay (LoS), type of surgical stabilization (open versus percutaneous surgical procedure), general in-hospital complications (cardiovascular events, deep venous thrombosis, pulmonary embolism, urinary tract infections, pneumonia, bedsore), surgery-related complications (haematoma, surgical site infection, neurological damage), revision surgeries and in-hospital mortality. Lumbopelvic fixation and every plate and screw osteosynthesis were regarded as open procedures; all other procedures were regarded as percutaneous. Patients, who received percutaneous procedures only, were defined as belonging to the P-group, patients who received a combination of an open and a percutaneous procedure and patients who received open procedures only were defined as belonging to the O-group. Radiographic images were analysed for implant malposition, implant loosening or implant failure. Living environment before hospital admission, mobility at discharge (ward, room, transfer, bedridden) and destination at discharge (home, geriatrics, rehabilitation, other hospital) were documented as well. Patients, who were able to walk in the room or on the floor were defined as “walkers”, patients who were bedridden or only able to perform transfers from bed to chairs as “non-walkers”. Patients, who returned home were regarded as “independent”, the others as “dependent”.

At follow up, patients or their relatives were contacted by phone and asked to participate in a survey. Their general practitioner or the bureau of vital statistics was contacted to ask about vital status, if patients were not directly available. All included patients or their relatives gave their oral approval for data analysis and participation in the survey. Actual quality of life (QoL), mobility and independence were graded with the Short Form-8 Physical Component Score (SF-8 PCS, range from 9.12 to 68.98) and Short Form-8 Mental Component Score (SF-8 MCS, range from 33.92 to 73.00) [19]. The mobility was further specified by the Parker Mobility Score (PMS), ranging from 0 to 9, higher scores equal to better mobility [20]. Subjective sensation of actual pain was rated with the numeric rating scale (NRS), scores from 0 to 10, higher scores indicating heavier pain [21]. The study was approved by the local ethics committee (Ethics Commission of the State Chamber of Medicine in Rhineland-Palatinate (Reference: 837.140.17 (10974))).

We tested continuous data for normal distribution using the Kolmogorov–Smirnov test. Descriptive statistics in normally distributed data were described as mean and standard deviation. In non-normally distributed data, median and the 25th and 75th interquartile ranges (IQR) were calculated. Different groups were compared using the non-paired student’s *t* test (normally distributed data) and the Mann–Whitney *U* test (non-normally distributed data). Nominal groups were compared using the chi-square test. Survival analysis was computed according to Kaplan–Meier. A *p* value of ≤ 0.05 was considered significant. Statistical analysis was performed using SPSS software (IBM SPSS Statistics for Windows, Version 23; IBM Corp, Armonk, NY, USA).

## Results

### Demographics

140 out of 500 patients (28%) were treated operatively, the others (72%) non-operatively (see flowchart in Table 1). There were 125 women (89.3%) and 15 men (10.7%). Sex distribution did not differ significantly between the subgroups (*p* = 0.69). Mean age was 77.4 years (SD 9.9 years)) Age did not differ significantly between the subgroups (*p* = 0.37). The youngest patient was only 39 year old. She sustained an infantile brain damage, had very limited mobility and received a long-term cortisone therapy. In relation to the total number of admitted patients, 2/138 with FFP I (1.4%), 52/238 with FFP type II (28%), 18/30 with FFP type III (60%) and 68/94 with FFP type IV (72.3%) were treated operatively. 129 patients (92.1%) presented with at least one, 69 patients (49.3%) with two or more comorbidities. A comorbidity was only registered as such when the disease was mentioned in the medical history of the discharge letter of the patient. Cardiovascular diseases, osteoporosis and malignancy were the most important concomitant diseases. The frequency of at least one or two and more comorbidities among FFP-classes was not significantly different (*p* values 0.75 resp. 0.78). Further details are given in Table 2.

**Table 2:** Demographics and comorbidities of all patients, depending on FFP-classification*

FFP type	FFP I–IV	FFP type I	FFP type II	FFP type III	FFP type IV	*p* value
Total number of patients	500	138	238	30	94	
Number of operated patients (*n*, %)	140 (28)	2 (1.4)	52 (21.8)	18 (60)	68 (72.3)	
Mean age (years, SD)	77.4 (9.9)	69.0 (10.0)	78.7 (8.1)	78.6 (11.9)	76.3 (10.2)	0.37
Women (*n*, %)	125 (89.3)	2	45 (86.5)	16 (88.9)	62 (91.2)	0.69
Men (*n*, %)	15 (10.7)	0	7 (13.5)	2 (11.1)	6 (8.8)	0.69
Patients with comorbidities (*n*, %)	129 (92.1)	2	49 (94.2)	17 (94.4)	61 (89.7)	0.75
Patients with two or more comorbidities (*n*, %)	69 (49.3)	2	27 (51.9)	9 (50.0)	31 (45.6)	0.78
Cardiovascular disease (*n*, %)	120 (85.7)	2	48 (92.3)	15 (83.3)	55 (80.9)	0.21
Osteopororsis (*n*, %)	90 (64.3)	2	32 (61.5)	10 (55.6)	46 (67.6)	0.58
Malignancy (*n*, %)	32 (22.9)	1	8 (15.4)	4 (22.2)	19 (27.9)	0.26
Diabetes mellitus (*n*, %)	27 (19.3)	0	15 (28.8)	4 (22.2)	8 (11.8)	0.07
Dementia (*n*, %)	12 (8.6)	0	4 (7.7)	2 (11.1)	6 (8.8)	0.84
Pulmonary disease (*n*, %)	17 (12.1)	2	6 (11.5)	2 (11.1)	7 (10.3)	1
Rheumatoid arthritis (*n*, %)	9 (6.4)	0	5 (9.6)	0	4 (5.9)	0.45

*P* values below 0.05 are shown in bold

*Due to the low number of patients with FFP Type I, % is not calculated

### Operative procedure

**Fig. 2:**
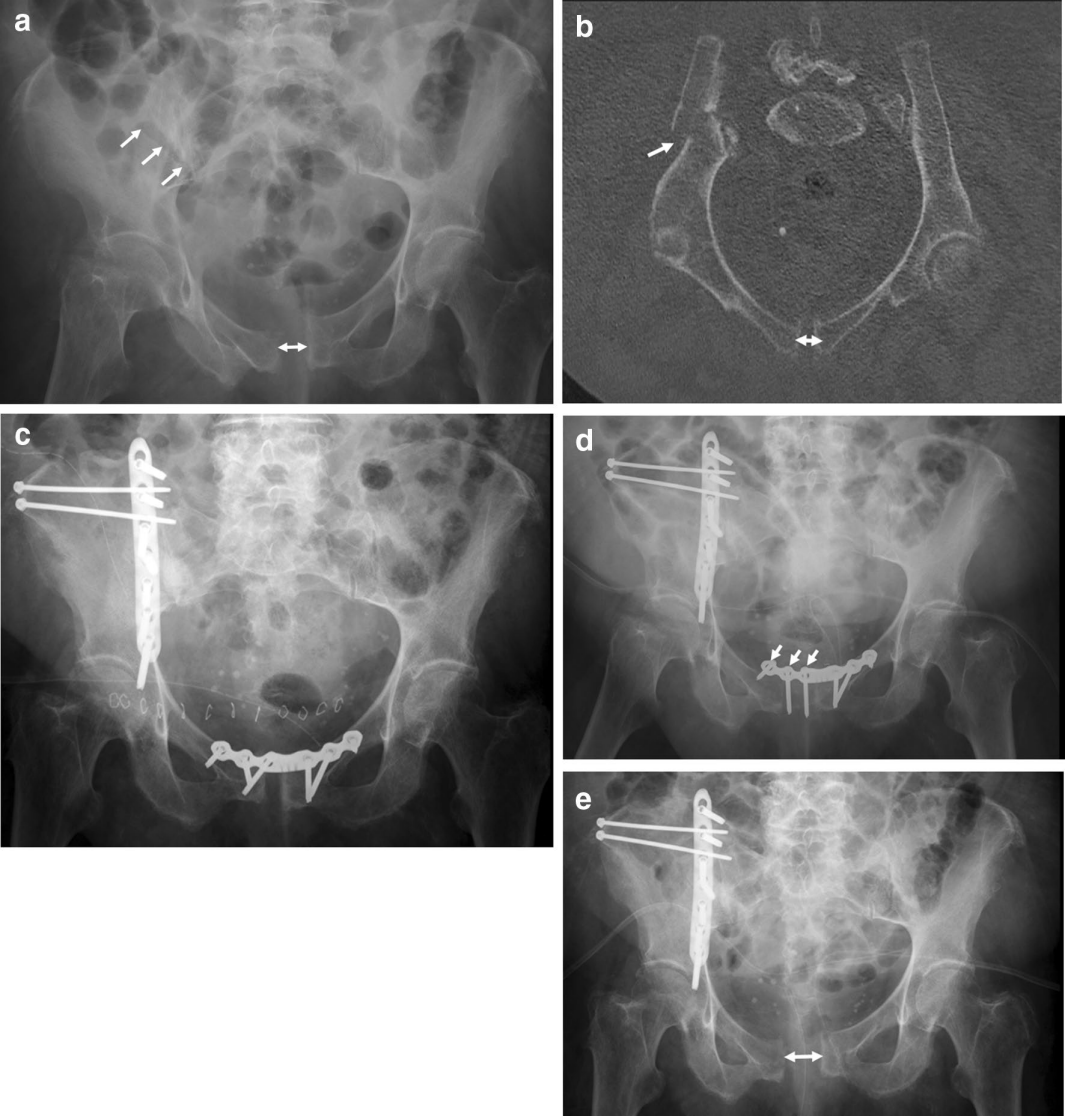
A 92-year-old female suffered a fall at home. The pelvic a.-p. overview shows a diastasis of the pubic symphysis and a fracture line at the right ilium (arrows) (**a**). CT-reconstruction along the pelvic brim shows the fracture of the right ilium and the diastasis of the pubic symphysis (arrows). The patient has a FFP type IIIa (**b**). Postoperative a.-p. pelvic overview. The ilium fracture and the pubic diastasis have been treated with open reduction and plate and screw osteosynthesis (**c**). Pelvic a.-p. overview two weeks after surgery. The three right screws of the pubic plate osteosynthesis show loosening. There are signs of surgical site infection. The symphysis plate needs to be removed and serial debridement becomes necessary (**d**). A.-p. pelvic overview after one month. The pubic diastasis has recurred. Due to surgical site infection at the ilium, serial debridement of the wound at the ilium is also needed (**e**)

The techniques of stabilization of the posterior and anterior pelvis (open versus percutaneous), depending on FFP-classification, are depicted in Table 3. All patients with FFP type III and IV received a posterior stabilization. The most frequently used technique for the posterior pelvis (as single procedure or in combination with other implants for the posterior pelvis, *n* = 83/136 procedures = 61.0%) was the trans-sacral bar osteosynthesis [22–24]. The rate of anterior stabilizations was 51.4% in patients with type FFP IV, 75% in patients with FFP Type II and 77.8% in patients with FFP Type III (*p* = 0.01). The most frequently used technique for the anterior pelvis (as single procedure or in combination with other implants for the anterior pelvis, *n* = 60/90 procedures = 66.7%) was the retrograde transpubic screw osteosynthesis (*p* < 0.001) [25]. Plate and screw osteosynthesis of the ilium was most often used in patients with FFP Type III (*p* = 0.02). A combination of osteosynthesis of the posterior and anterior pelvis was performed in 86 of 140 patients (61.4%). 99 patients (70.7%) received percutaneous procedures only (P-group), 41 patients (29.3%) received open procedures with or without an additional percutaneous procedure (O-group). Patients with FFP Type III had the highest rate of open procedures (66.7%), followed by patients with FFP Type IV (32.4%) and FFP Type II (11.5%) (*p* < 0.001) (Fig. 2a–e).

**Table 3:** Frequency and type of operative stabilization of the posterior and anterior pelvis, depending on FFP-classification

FFP categories		FFP I–IV	FFP type I	FFP type II	FFP type III	FFF type IV	*p* value
Number of operatively treated patients		140	2	52	18	68	
Posterior pelvis	O or P						
IS screws unilateral	P	30	0	25	3	2	** < 0.001**
IS screws bilateral	P	7	0	2	1	4	0.88
IS screw with plate and screw osteosynthesis ilium	O	2	0	0	1	1	0.24
Transsacral bar	P	29	0	9	1	19	0.09
Transsacral bar with unilateral IS screw	P	16	0	7	3	6	0.46
Transsacral bar with bilateral IS screws	P	36	0	6	0	30	** < 0.001**
Transsacral bar with plate and screw osteosynthesis ilium	O	2	0	0	0	2	0.63
Transiliac bridging plate osteosynthesis with bilateral IS screws	P	1	0	0	0	1	1
Plate and screw osteosynthesis ilium	O	10	0	0	9	1	** < 0.001**
Internal fixator	P	1	0	1	0	0	0.51
Internal fixator with bilateral IS screws	P	1	0	0	0	1	1
Lumbopelvic fixation with bilateral IS screws	O	1	0	0	0	1	1
Sum		136	0	50	18	68	0.38
Number of operatively treated patients		140	2	52	18	68	
Anterior pelvis	O or P						
Unilateral retrograde transpubic screw	P	48	0	29	6	13	** < 0.001**
Bilateral retrograde transpubic screw	P	9	1	4	1	3	0.78
Retrograde transpubic screw and plate and screw osteosynthesis	O	3	0	1	0	2	1
Plate and screw osteosynthesis	O	28	1	5	7	15	**0.02**
Plate and screw osteosynthesis and external fixator	O	2	0	0	0	2	0.63
Sum		90	2	39	14	35	0.01
Patients who received percutaneous procedures only (P-group)(*n*, %)	P	99 (70.7)	1	46 (88.5)	6 (33.3)	46 (67.6)	** < 0.001**
Patients who received open procedures (O-group) (*n*, %)	O	41 (29.3)	1	6 (11.5)	12 (66.7)	22 (32.4)	** < 0.001**

*P* values below 0.05 are shown in bold

*O* open procedure, *P* percutaneous procedure

*Due to the low number of patients with FFP type I, % is not calculated

### Postoperative course

The median LoS was 18 days (3–92 days, IQR 14–25 days). Surgical stabilization was performed at a median of 6 days after admission. The median postoperative LoS was 12 days (2–74 days, IQR 9–17 days). There was no significant difference between the subgroups (*p* = 0.3) but there was a significant difference between the P-group and the O-group (*p* = 0.009). There was no in-hospital mortality. Table 4 demonstrates the type and frequency of general in-hospital and (early and late) surgery-related complications and the number of surgical revisions needed. During hospital stay, 51 patients (36.4%) suffered general complications, the most frequent being urinary tract infection and bedsores (all of them without skin necrosis). 37 patients (26.4%) suffered surgery-related complications, the most frequent being surgical site haematoma and implant loosening. The distribution among the subgroups was not significantly different (*p* = 0.15). 25 patients (17.9%) needed surgical revisions without difference between the subgroups (*p* = 0.78). Patients with FFP Type III had the most often open surgical procedures (66.7%), suffered the most frequent general (66.7%) and surgery-related complications (44.4%) and had the longest postoperative LoS (14.5 days).

**Table 4:** Postoperative course of all patients, depending on FFP-classification*

	FFP type I–IV	FFP type I	FFP type II	FFP type III	FFP type IV	*p* value
All patients (*n*)	140	2	52	18	68	
Open procedures (*n*; %)	41 (29.3)	1	6 (11.5)	12 (66.7)	22 (32.4)	** < 0.001**
Median LoS (days)	18	9.5	17	21	18	0.65
Minimum (days)	3	9	3	10	6	
Maximum (days)	92	10	92	56	68	
IQR (days)	14–25	9.25–9.75	14.8–25.0	14.5–24.5	13–25.5	
Median postoperative LoS (days)	12	6	12	14.5	12	0.3
Minimum (days)	2	2	3	8	3	
Maximum (days)	74	10	74	49	51	
IQR (days)	9–17	4–8	9–15	11.25–17.75	9–18	
Patients with general in-hospital complications (*n*, %)	51 (36.4)	0	14 (26.9)	12 (66.7)	24 (35.3)	**0.01**
Urinary tract infection (UTI) (*n*, %)	37 (26.4)	0	10 (19.2)	9 (50.0)	18 (26.5)	**0.046**
Pneumonia (*n*, %)	7 (5.0)	0	3 (5.8)	1 (5.6)	3 (4.4)	1
Cardiovascular (*n*, %)	4 (2.9)	0	3 (5.8)	0 (0.0)	1 (1.5)	0.35
Bedsore (without skin necrosis) (*n*, %)	11 (7.9)	0	5 (9.6)	3 (16.7)	3 (4.4)	0.13
Thrombosis (*n*, %)	4 (2.9)	0	2 (3.8)	0 (0.0)	2 (2.9)	1
Lung embolism (*n*, %)	2 (1.4)	0	1 (1.9)	1 (5.5)	0 (0.0)	0.12
Patients with surgery-related complications (*n*, %)	37 (26.4)	1	11 (21.2)	8 (44.4)	17 (25)	**0.15**
Hematoma (*n*, %)	10 (7.1)	0	3 (5.8)	1 (5.6)	6 (8.8)	0.90
Infection (*n*, %)	7 (5)	0	3 (5.8)	2 (11.1)	2 (2.9)	0.29
Implant malposition (*n*, %)	2 (1.4)	0	2 (3.8)	0	0	0.38
Implant loosening (*n*, %)	13 (9.3)	0	4 (7.7)	2 (11.1)	7 (10.3)	0.85
Implant failure (*n*, %)	3 (2.1)	0	1 (1.9)	1 (5.6)	1 (1.5)	0.51
Dysesthesia–paresis (*n*, %)	7 (5)	0	2 (3.8)	3 (16.7)	2 (2.9)	0.08
Suprapubic hernia (*n*, %)	2 (1.4)	1	1 (1.9)	0	0	0.51
Patients with surgical revision (%)	25 (17.9)	1	9 (17.3)	4 (22.2)	11 (16.2)	0.78

*P* values below 0.05 are shown in bold

*LoS* Length of hospital Stay

*Due to the low number of patients with FFP type I, % is not calculated

### Percutaneous versus open procedures

99 patients (70.7%) belonged to the P-group, 41 patients (29.3%) to the O-group. Table 5 gives a comparison between the demographics and complications of the P-group and the O-group. There were no significant differences in the demographics. There were more surgery-related complications (*p* = 0.006), more surgical revisions (*p* = 0.02) and there was a longer postoperative stay (*p* = 0.009) in the O-group. The number of general in-hospital complications was not different between the groups (*p* = 0.72).

**Table 5:** Demographics and postoperative course of all patients, depending on type of treatment (P-group versus O-group)

Type of treatment	All patients	P-group	O-group	*p* value
Number of patients (%)	140 (100)	99 (70.7)	41 (29.3)	
Mean age (years, SD)	77.4 (10.7)	77.6 (10.2)	77.0 (8.9)	0.78
Women (*n*, %)	125 (89.3)	89 (89.9)	36 (87.8)	0.77
Men (*n*, %)	15 (10.7)	10 (10.1)	5 (12.2)	0.77
Patients with comorbidities (*n*, %)	129 (92.1)	91 (91.9)	38 (92.7)	1
Patients with two or more comorbidities (*n*, %)	69 (49.3)	50 (50.5)	19 (46.3)	0.65
Median LoS (days)	18	17	20	0.12
Minimum (days)	3	3	9	
Maximum (days)	92	72	92	
IQR (days)	14–25	14–25	14–28	
Median LoS postoperative (days)	12	12	14	**0.009**
Minimum postoperative (days)	2	2	7	
Maximum postoperative (days)	74	65	74	
IQR (days)	9–17	8.5–15	10–21	
Patients with general complications (*n*, %)	51 (36.4)	37 (37.4)	14 (34.1)	**0.72**
Urinary tract infection (*n*, %)	37 (26.4)	26 (26.3)	11 (26.8)	0.94
Pneumonia (*n*, %)	7 (5.0)	6 (6.1)	2 (4.9)	1
Cardiovascular (*n*, %)	4 (2.9)	4 (4.0)	0 (0.0)	0.32
Bedsore (*n*, %)	11 (7.9)	7 (7.1)	4 (9.8)	0.73
Thrombosis (*n*, %)	4 (2.9)	3 (3.0)	1 (2.4)	1
Lung embolism (*n*, %)	2 (1.4)	0 (0.0)	2 (4.9)	0.08
Patients with surgical complications (%)	37 (26.4)	19 (19.2)	18 (43.9)	**0.006**
Hematoma (*n*, %)	10 (7.1)	7 (7.1)	3 (7.3)	1
Infection (*n*, %)	7 (5)	4 (4.0)	3 (7.3)	0.42
Implant malposition (*n*, %)	2 (1.4)	1 (1.0)	1 (2.4)	0.5
Implant loosening (*n*, %)	13 (9.3)	7 (7.1)	6 (14.6)	0.2
Implant failure (*n*, %)	3 (2.1)	0	3 (7.3)	**0.02**
Dysesthesia–paresis (*n*, %)	7 (5)	3 (3.0)	4 (9.8)	0.19
Suprapubic hernia (*n*, %)	2 (1.4)	0	2 (4.9)	0.08
Patients with surgical revisions (%)	25 (17.9)	13 (13.1)	12 (29.3)	**0.02**

*P* values below 0.05 are shown in bold

### Mobility and destination at discharge

Patients were discharged from hospital after a median of 12 postoperative days. Patients with FFP Type I had shortest postoperative LoS (6.0 days), patients with FFP Type III had the longest (14.5 days). Table 6 gives an overview of the mobility at discharge, depending on FFP-classification and type of treatment (P-group versus O-group). There was no significant difference between the FFP-classes (*p* = 0.47) and the type of treatment (*p* = 0.13). The lowest rate of “walkers” was observed in patients with FFP Type III (44.4%) and in the O-group (46.3%). Table 7 compares the living environment before admission and their destination at discharge of all patients, depending on FFP-classification and on type of treatment (P-group and O-group). There is a dramatic decrease of independency at discharge in the whole group (85.4% versus 28.6%) and in all subgroups (*p* < 0.001).

**Table 6:** Mobility at discharge of all patients, depending on FFP-classification and type of treatment (P-group versus O-group)

FFP type	FFP type I–IV	FFP type I	FFP type II	FFP type III	FFP type IV	*p* value
All patients	140	2	52	18	68	
Mobility at discharge not documented	6	0	3	0	3	
Documented patients (*n*, %)	134 (100)	2	49 (100)	18 (100)	65 (100)	
Ward (*n*, %)	54 (40.3)	1	22 (44.9)	5 (27.8)	26 (40.0)	0.45
Room (*n*, %)	21 (15.7)	0	8 (16.3)	3 (16.7)	10 (15.4)	1
Transfer (*n*, %)	52 (38.8)	1	18 (36.7)	9 (50.0)	24 (36.9)	0.57
Bed (*n*, %)	7 (5.2)	0	1 (2.1)	1 (5.6)	5 (7.7)	0.35
Walkers (*n*, %)	75 (56.0)	1	30 (61.2)	8 (44.4)	36 (55.4)	0.47
Non-walkers (*n*, %)	59 (44.0)	1	19 (38.8)	10 (55.6)	29 (44.6)	0.47

Type of treatment	All patients	P-group	O-group	*p* value
All patients	140	99	41	
Mobility at discharge not documented	6	6	0	
Documented patients (*n*, %)	134 (100)	93 (100)	41 (100)	
Ward (*n*, %)	54 (40.3)	39 (41.9)	15 (36.6)	0.76
Room (*n*, %)	21 (15.7)	17 (18.3)	4 (9.8)	0.31
Transfer (*n*, %)	52 (38.8)	35 (37.6)	17 (41.5)	0.5
Bed (*n*, %)	7 (5.2)	2 (2.2)	5 (12.2)	**0.02**
Walkers (*n*, %)	75 (56.0)	56 (60.2)	19 (46.3)	0.13
Non-walkers (*n*, %)	59 (44.0)	37 (39.8)	22 (53.7)	0.13

*P* values below 0.05 are shown in bold

**Table 7:** Living environment before admission and destination at discharge from hospital of all patients, depending on FFP-classification and on type of treatment (P-group and O-group)

FFP type II–IV	Pre-hospital	Discharge	*p* value	FFP type II	Pre-hospital	Discharge	*p* value
All patients	138	138		All patients	52	52	
Not documented	8	5		Not documented	3	1	
Documented patients (*n*, %)	130 (100)	133 (100)		Documented patients (*n*, %)	49 (100)	51 (100)	
Home (*n*, %)	111 (85.4)	38 (28.6)	** < 0.001**	Home (*n*, %)	42 (85.7)	19 (37.3)	** < 0.001**
Geriatrics (*n*, %)	12 (9.2)	49 (36.8)	** < 0.001**	Geriatrics (*n*, %)	5 (10.2)	11 (21.6)	0.12
Rehabilitation (*n*, %)	2 (1.5)	38 (28.6)	** < 0.001**	Rehabilitation (*n*, %)	1 (2.0)	18 (35.3)	** < 0.001**
Hospital (*n*, %)	5 (3.8)	8 (6.0)	0.42	Hospital (*n*, %)	1 (2.0)	3 (5.9)	0.61
*p* value for all subgroups			** < 0.001**	*p* value for all subgroups			** < 0.001**
Independent (*n*, %)	111 (85.4)	38 (28.6)		Independent (*n*, %)	42 (85.7)	19 (37.3)	
Dependent (*n*, %)	19 (14.6)	95 (71.4)	** < 0.001**	Dependent (*n*, %)	7 (14.3)	32 (62.7)	** < 0.001**

FFP type III	Pre-hospital	Discharge	*p* value	FFP type IV	Pre-hospital	Discharge	*p* value
All patients	18	18		All patients	68	68	
Not documented	1	0		Not documented	4	4	
Documented patients (*n*, %)	17 (100)	18 (100)		Documented patients (*n*, %)	64 (100)	64 (100)	
Home (*n*, %)	16 (94.1)	7 (38.9)	** < 0.001**	Home (*n*, %)	53 (82.8)	12 (18.8)	** < 0.001**
Geriatrics (*n*, %)	1 (5.9)	7 (38.9)	**0.04**	Geriatrics (*n*, %)	6 (9.4)	31 (48.4)	** < 0.001**
Rehabilitation (*n*, %)	0	3 (16.7)	0.23	Rehabilitation (*n*, %)	1 (1.6)	17 (26.6)	** < 0.001**
Hospital (*n*, %)	0	1 (5.6)	1	Hospital (*n*, %)	4 (6.2)	4 (6.2)	1
*p* value for all subgroups			**0.003**	*p* value for all subgroups			** < 0.001**
Independent (*n* %)	16 (94.1)	7 (38.9)		Independent (*n*, %)	53 (82.8)	12 (18.8)	
Dependent (*n* %)	1 (5.7)	11 (61.1)	** < 0.001**	Dependent (*n*, %)	11 (17.2)	52 (81.2)	** < 0.001**

P-group	Pre-hospital	Discharge	*p* value	O-group	Pre-hospital	Discharge	*p* value
All patients	99	99		All patients	41	41	
Not documented	5	4		Not documented	3	1	
Documented patients	94 (100)	95 (100)		Documented patients (%)	38 (100)	40 (100)	
Home (*n*, %)	78 (83.0)	28 (29.5)	** < 0.001**	Home (*n*, %)	35 (92.1)	11 (27.5)	** < 0.001**
Geriatrics (*n*, %)	10 (10.6)	31 (32.6)	** < 0.001**	Geriatrics (*n*, %)	2 (5.3)	18 (45.0)	** < 0.001**
Rehabilitation (*n*, %)	2 (2.1)	31 (32.6)	** < 0.001**	Rehabilitation (*n*, %)	0 (0.0)	8 (20.0)	**0.005**
Hospital (*n*, %)	4 (4.3)	5 (5.3)	1	Hospital (*n*, %)	1 (2.6)	3 (7.5)	0.62
*p* value for all subgroups			** < 0.001**	*p* value for all subgroups			** < 0.001**
Independent (*n*, %)	78	28		Independent (*n*, %)	35	11	
Dependent (*n*, %)	16	67	** < 0.001**	Dependent (*n*, %)	3	29	** < 0.001**

*P* values below 0.05 are shown in bold

### Follow-up

One-year mortality was 9.7%, 2-year mortality 15.7% and 5-year mortality 41.5%. There was no influence of FFP-classes or type of treatment on mortality (*p* = 0.428 resp. 0.831). 91 patients could be reached via telephone call for the survey, which corresponds with a follow up of 85.8% of surviving patients. Median follow-up time was 40 months. Median SF-8 physical component score (PCS) was 32.43 (min. 17.34–max. 57.32), median SF-8 mental component score (MCS) was 54.42 (min. 17.71–max. 69.22). There was no influence of the FFP-classes or the type of treatment. The highest SF-8 PCS and SF-8 MCS values were found in the FFP Type I subgroup, the lowest in the FFP Type III subgroup. Median PMS for all patients was 5 (min. 0–max. 9). The lowest values were found in the FFP Type III subgroup and in the O-group. Median NRS for all patients was 4 (min. 0–max. 10). Further data are shown in Table 8.

**Table 8:** Mortality and follow up of all patients, depending on FFP-classification and on type of treatment (P-group versus O-group)

FFP-type	FFP Type I-IV	FFP Type I	FFP Type II	FFP Type III	FFP Type IV	p-value
Number of operated patients	140	2	52	18	68	
One-year mortality (%)	9.7	0	8	12.5	11.8	
Two-year mortality (%)	15.7	0	8	26	19.5	
Five-year mortality (%)	41.5	0	33.2	26	51	
Overall mortality						0.428
Patients who died before follow up (%)	34 (24.3)	0	11 (21.2)	5 (27.8)	18 (26.5)	
Patients lost to follow up (%)	15 (10.7)	0	6 (11.5)	2 (11.1)	7 (10.3)	
Patients with follow-up (%)	91 (65.0)	2	35 (67.3)	11 (61.1)	43 (63.2)	
Surviving patients with follow up (%)	91 (85.8)	2	35 (85.4)	11 (84.6)	43 (86.0)	
Median follow up time (months)	40	42	42	47	39	
**Patients with SF-8 (n, %)**	60/91 (65.9)	2/2	16/35 (45.7)	5/11 (45.5)	37/43 (86.0)	
Median SF-8 physical (PCS)	32.43	46.12	29.39	27.88	32.88	0.61
Min SF-8 physical	17.34	34.92	17.34	22.54	18.59	
Max SF-8 physical	57.32	57.32	56.68	42.43	55.50	
Median SF-8 mental (MCS)	54.42	56.50	55.42	50.21	54.67	0.32
Min SF-8 mental	17.71	53.93	19.83	17.85	17.71	
Max SF-8 mental	69.22	59.08	65.17	58.61	69.22	
**Patients with PMS (n, %)**	91/91 (100.0)	2/2	35/35 (100.0)	11/11 (100.0)	43/43 (100.0)	
Median PMS	5	6.5	6	3	5	0.20
Min PMS	0	4	0	0	0	
Max PMS	9	9	9	9	9	
**Patients with NRS (n, %)**	89/91 (97.8)	2/2	35/35 (100.0)	10/11 (90.9)	42/43 (97.7)	
Median NRS	4	0	5	4	4	0.86
Min NRS	0	0	0	0	0	
Max NRS	10	0	9	7	10	
**Type of treatment**	**All patients**	**P-group**	**O-group**	**p value**		
Number of operated patients	140	99	41			
One-year mortality (%)	9.7	11.5	7.7			
Two-year mortality (%)	15.7	12.8	23.7			
Five-year mortality (%)	41.5	43.3	32.4			
Overall mortality				0.831		
Patients who died before follow up (%)	34 (24.3)	26 (26.3)	8 (19.5)			
Patients lost to follow up (%)	15 (10.7)	13 (13.1)	2 (4.9)			
Patients with follow-up (%)	91 (65.0)	60 (60.6)	31 (75.6)			
Surviving patients with follow up (%)	91 (85.8)	60 (82.2)	31 (93.9)			
Median follow up time (months)	40	39.6	40.5			
**Patients with SF-8 (n, %)**	60/91 (65.9)	38/60 (63.3)	22/31 (71.0)			
Median SF-8 physical (PCS)	32.43	32.64	31.22	0.68		
Min SF-8 physical	17.34	17.34	18.59			
Max SF-8 physical	57.32	56.68	57.32			
Median SF-8 mental (MCS)	54.42	54.26	58.82	0.22		
Min SF-8 mental	17.71	17.71	17.85			
Max SF-8 mental	69.22	67.27	69.22			
**Patients with PMS (n, %)**	**91/91 (100.0)**	60/60 (100.0)	31/31 (100.0)			
Median PMS	5	6	4	0.07		
Min PMS	0	0	0			
Max PMS	9	9	9			
**Patients with NRS (n, %)**	**89/91 (97.8)**	59/60 (98.3)	30/31 (96.8)			
Median NRS	4	3	4	0.30		
Min NRS	0	0	0			
Max NRS	10	9	10			

P-values below 0.05 are shown in bold

## Discussion

Fragility fractures of the pelvis are associated with intense and immobilizing pain. Main goals of treatment are early, pain-free mobilization and restoration of previous patient self-sufficiency. In many patients, these objectives can be reached by conservative treatment [11, 12]. To date, indications for and type of operative treatment are still a matter of discussion [16, 26]. In this retrospective study, we analysed the risks and benefits of operative treatment in 140 patients, depending on FFP-classification and surgical invasivity. To the best of our knowledge, no other study analysed operative management of FFP in such detail.

The demographics of our patients are similar to those of comparable case series in literature: median age is around 80 years and the vast majority of them are women [9, 27–30]. Our most important finding is that patients of the O-group suffered more often from complicated, non-fatal postoperative conditions than patients, who received percutaneous procedures. Patients of the O-group had significantly more surgical complications (*p* = 0.006), needed more often surgical revisions (*p* = 0.02) and also had a the longest postoperative LoS (14 days) (*p* = 0.009). These findings were the most obvious in the patients with FFP Type III, of whom 66.7% underwent an open surgical procedure: 66.7% suffered general complications, 44.4% surgery-related complications and 22.2% needed surgical revisions. The patients of this subgroup also had the longest LoS (21 days) and the longest postoperative LoS (14.5 days). Although 94.1% lived at home before hospital admission, only 44.4% were walkers and 38.9% independent at discharge. At follow up, they also showed the lowest SF-8 PCS (27.88), the lowest SF-8 MCS (50.21) and the lowest PMS (3). Our results are similar to those of Gericke et al. [17]. In their study, patients with an open procedure had a twofold surgery-related complication rate than patients with a percutaneous procedure (18.1% vs. 9.5%), but did not have a higher rate of general complications. LoS was the highest in the group with open operative procedure [17].

**Fig. 3:**
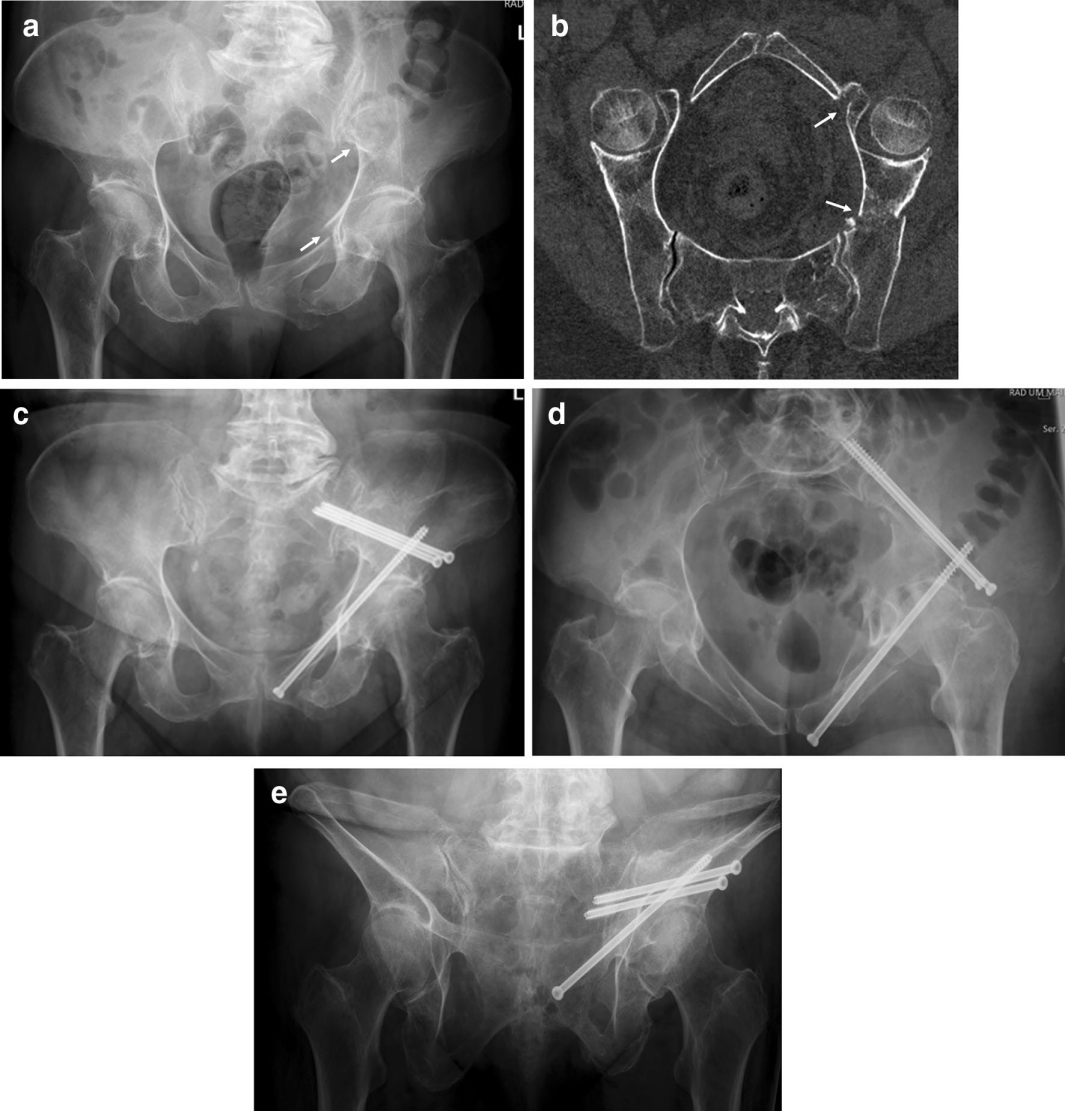
A 79-year-old female suffered a fall at home. The pelvic a.-p. overview reveals a displaced fracture of the left upper and lower pubic ramus and a displaced fracture of the left ilium (arrows) (**a**). CT-reconstruction along the pelvic brim shows the fracture of the left ilium and of the superior pubic ramus near to the anterior lip of the acetabulum (arrows). The patient has a FFP type IIIa (**b**). Pelvic a.-p. overview 6 months after operation. The ilium fracture was stabilized with two supra-acetabular screws from the anterior inferior to the posterior superior iliac spine. The pubic ramus fracture was stabilized with a retrograde transpubic screw. The screw insertions were performed percutaneously (**c**). Pelvic inlet view (**d**). Pelvic outlet view (**e**). The patient is able to walk independently up to 30 minutes. PMS is 9

Patients with FFP are of high age and have comorbidities, which put them at risk for in-hospital complications. Surgical treatment may be responsible for additional morbidity and mortality. It underlines the need for a critical, patient-specific evaluation of every indication for surgery and underlines the importance of minimal-invasive procedures, when surgery is indicated [31, 32]. Open surgical procedures should be avoided, if not clearly needed. Slight fracture displacements do not need open reduction but reliable stabilization [33]. Alternative minimal-invasive surgical procedures for stabilization of ilium fractures have been described recently (Fig. 3a–e) [34–36]. Plate and screw osteosynthesis of the anterior pelvic ring also proved to be complicated in our patient group. Besides the risks, connected to any open procedure, there is a high risk of implant loosening or breakage [37]. Minimal-invasive stabilization techniques have been developed for the anterior pelvis, although results of large series in the elderly population are not available to date [38–41]. Further biomechanical work and feasibility studies are needed to find the most reliable solution.

Fifty-one patients (36.4%) suffered from general, but not life-threatening complications during hospital stay. Urinary tract infection, bedsores and pneumonia are typical in-hospital acquired complications of bedridden conditions. A shorter preoperative LoS, which was 6 days in our series, may reduce this rate. For comparison, complication rate of 138 patients with FFP type I with a LoS of 8 days was only 16.5% [18]. In our study, the rate of general complications did not differ between the P-group and the O-group (*p* = 0.72) but was significantly higher in patients with FFP type III, who had the longest LoS (*p* = 0.01). Gericke et al. also found out that patients with open surgical procedures did not have more general complications than patients with percutaneous procedures (33.0% versus 28.4%). In their listing, delirium was mentioned but bedsores not [17]. van Dyck et al. registered 20.2% of in-hospital complications, of which urinary tract infection and pneumonia also were the most frequent [27]. Banierink et al. calculated a complication rate of 23% within 30 days after injury [28]. Both studies report on patients, who were treated conservatively (100% resp. 92%) [27, 28].

One-year mortality was 9.7% for the whole group. Patients with FFP Type III had the highest 1-year mortality (12.5%). There was no significant difference between the FFP-classes and the types of treatment. These rates are much lower than the rates, presented in other series: Banierink published a one-year mortality of 26.8% [28], van Dijk 24.7% [27], Loggers 23% [42], Andrich 21% [43], Osterhoff 23% of operative and 17% of non-operative patients [16]. Only Yoshida published a one-year mortality of 6.7% [12]. Our low mortality rate study is still double as in a reference population of the same state, where one-year mortality is 5.9% for men and 4% for women [44]. Andrich published a one-year mortality of 11% among 193.159 patients of the same age without pelvic fracture [43]. Our data point out that operatively treated patients with FFP show low mortality rates, despite a complicated perioperative course.

SF-8 PCS and SF-8 MCS and PMS did not differ significantly between the FFP-classes or the types of treatment. All values were moderate to low, which indicates the important loss of mobility and independency after FFP. SF-8 PCS is moderate for all patients with a value of 32.43. The lowest values were found in patients with FFP Type III, who also scored worst for other parameters. Only a minority of all operated patients lived without pain. Median NRS (range 0–10) was 4.

## Conclusion

In this retrospective study, we critically analysed the consequences of operative treatment in 140 patients with FFP. Patients who received an open surgical procedure had a longer postoperative LoS and suffered more surgery-related complications. They also needed more surgical revisions. At discharge, there was a significant drop in mobility and independency in patients of all categories. At follow up, all patients showed moderate physical and mental component scores, restricted mobility and moderate pain. The FFP-classes or the invasivity of treatment did not influence these follow up scores. Mortality was lower than in similar series, published in literature. Prospective studies are needed to further identify the optimal indications for and best techniques of operative treatment.
